# RNADSN: Transfer-Learning 5-Methyluridine (m^5^U) Modification on mRNAs from Common Features of tRNA

**DOI:** 10.3390/ijms232113493

**Published:** 2022-11-04

**Authors:** Zhirou Li, Jinge Mao, Daiyun Huang, Bowen Song, Jia Meng

**Affiliations:** 1 School of AI and Advanced Computing, Xi’an Jiaotong-Liverpool University, Suzhou 215123, China; 2 Department of Biological Sciences, Xi’an Jiaotong-Liverpool University, Suzhou 215123, China; 3 Department of Computer Science, University of Liverpool, Liverpool L69 7ZB, UK; 4 Institute of Systems, Molecular and Integrative Biology, University of Liverpool, Liverpool L69 7ZB, UK; 5 AI University Research Centre, Xi’an Jiaotong-Liverpool University, Suzhou 215123, China

**Keywords:** 5-methyluridine, deep neural network, transfer learning, RNA modification, site prediction

## Abstract

One of the most abundant non-canonical bases widely occurring on various RNA molecules is 5-methyluridine (m5U). Recent studies have revealed its influences on the development of breast cancer, systemic lupus erythematosus, and the regulation of stress responses. The accurate identification of m^5^U sites is crucial for understanding their biological functions. We propose RNADSN, the first transfer learning deep neural network that learns common features between tRNA m^5^U and mRNA m^5^U to enhance the prediction of mRNA m^5^U. Without seeing the experimentally detected mRNA m^5^U sites, RNADSN has already outperformed the state-of-the-art method, m5UPred. Using mRNA m^5^U classification as an additional layer of supervision, our model achieved another distinct improvement and presented an average area under the receiver operating characteristic curve (AUC) of 0.9422 and an average precision (AP) of 0.7855. The robust performance of RNADSN was also verified by cross-technical and cross-cellular validation. The interpretation of RNADSN also revealed the sequence motif of common features. Therefore, RNADSN should be a useful tool for studying m^5^U modification.

## 1. Introduction

It has been demonstrated that more than 170 post-transcriptional RNA modifications are present within a diverse set of RNAs, most of which occur in the tRNA and rRNA [1]. These chemical modifications have been proven to be related to various biological functions, such as early embryonic development [2], cancer stem-cell-fate decisions [3,4], and brain neurodevelopment [5,6]. The accurate identification of RNA-modification sites is crucial for an in-depth understanding of the regulatory circuitry of RNA life in all species.

The past few years have witnessed an increasing number of computational approaches to predicting epigenetic RNA modifications that serve as useful alternatives to wet-laboratory experiments. These include iRNA toolkits [7,8,9,10], SRAMP [11], M6APred-EL [12], DeepPromise [13], WHISTLE [14], Gene2vec [15], NmSEER [16], m7G-IFL [17], RF-PseU [18], MultiRM [19], and DeepAc4C [20]. Special attention has also been paid to cross-species prediction [21,22,23,24], tissue-specific prediction [25,26,27], and learning from low-resolution data [28,29]. User-friendly databases [30,31], platforms [32,33,34,35], and tools [36,37] have also been developed. Together, these works greatly have advanced our understanding of RNA modifications.

Previous predictors focused solely on modifications from a single RNA type, while the coordination of tRNA and mRNA modifications has recently been reported [38,39], suggesting a new layer of regulation relationship that can be considered in the modeling. The tRNAs are prime targets for modification and have been extensively studied for decades. In recent years, mRNA molecules have also been shown to be heavily modified. Some modifications were reported in both tRNA and mRNA. In particular, pseudouridine (Ψ) and m^6^A were recently found to be potential common denominators between tRNA and mRNA. Some tRNA pseudouridine synthases were also found to modify mRNA [40] using similar RNA motifs [41]. A recent study showed that the ablation of the tRNA methyltransferase, TRMT10A, could increase mRNA m^6^A methylation levels by interacting with the m^6^A demethylase FTO. Unlike Ψ and m^6^A, which have been extensively studied, 5-methyluridine (m^5^U), another shared modification between tRNA and mRNA, is underexplored due to the limited high-throughput profiling approaches. Only recently did Carter et al. rely on the m^5^U-catalyzing enzyme, TRMT2A, to apply miCLIP and develop FICC-seq to detect m^5^U in human RNAs. FICC-seq successfully identified tRNA sites in a manner that was consistent with existing knowledge, while also revealing m^5^U sites on mRNAs. However, the relatively small number of reported mRNA sites compared to tRNA sites limits the development of computational models to predict sites from other conditions or samples. Inspired by the potential coordination of tRNA and mRNA modifications, in this study, we show that the computational identification of modifiable mRNA sites by learning from common tRNA features could be a promising approach.

We propose the RNA domain separation network (RNADSN), the first transfer-learning deep neural network to extract shared features between tRNA and mRNA m^5^U modifications and learn to predict human mRNA m^5^U sites. Current m^5^U predictors are constructed either for tRNA sites only [42] or for a mixture of tRNA and mRNA sites [43] and, thus, cannot reflect the true performance on mRNA. Motivated by the domain-separation network [44], RNADSN accepts both tRNA data and mRNA data and utilizes three networks to capture tRNA m5U-specific, mRNA m^5^U-specific, and shared features, respectively. Only these shared features are used, so that the model can capture the tRNA features with the best generalizability to predict mRNA sites. RNADSN achieved an area under a receiver operating characteristic curve (AUC) of 94.22% and an average precision (AP) of 78.55% for identifying mRNA m^5^U sites at a positive–negative ratio of 1:10, which was better than that of the baseline model m5UPred [43]. Cross-cellular and cross-technical evaluations also validated the robustness of our model. Furthermore, RNADSN allows motif mining through model interpretation to reveal captured shared-sequence patterns. Therefore, RNADSN can serve as a useful tool to identify mRNA m^5^U and study the coordination between tRNA and mRNA modifications.

## 2. Results and Discussion

### 2.1. RNADSN Allows Transfer Learning from tRNA m^5^U to mRNA m^5^U

**Figure 1:**
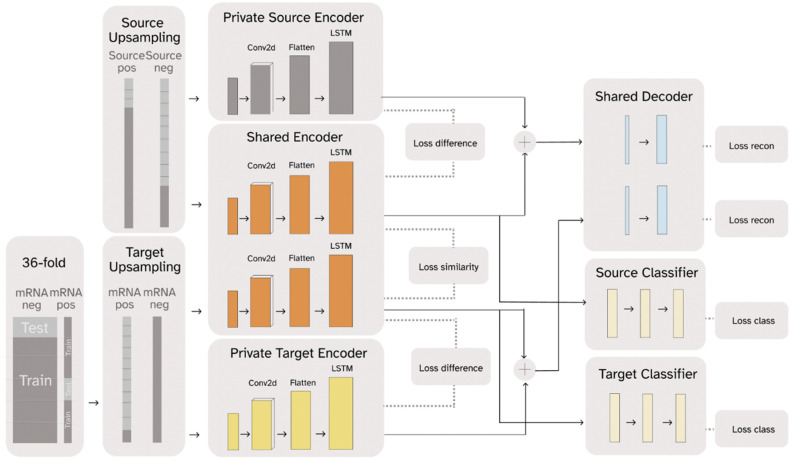
A simplified graphic illustration of the proposed RNADSN framework.

RNADSN, as shown in Figure 1**,** predicts mRNA m^5^U modification sites using transfer learning. The framework is enlightened by Domain Separation [44] and treats tRNA data as the source domain and mRNA data as the target domain. RNA sequences from each domain pass through a domain-specific private encoder and a shared encoder. For each domain, the outputs of these two encoders are combined again and trained to reconstruct the original input so that all the sequence features are split into the two encoders without information loss. At the same time, the model is optimized to minimize the difference between the two domain outputs in the shared encoder and maximize the difference between the private encoder and the shared encoder for each domain.

For m^5^U prediction, only the hidden representations extracted by the shared encoder are used to train the classifier. Since we had access to tRNA m^5^U data, both positive and negative tRNA data were sent to the source-domain encoders and used to train a tRNA m^5^U predictor so that the shared encoder could learn to capture m^5^U sequence patterns. Initially, we only used mRNA-negative data to train the target domain, so that the model only saw the mRNA sequence context and not the mRNA m^5^U-specific patterns. We found that our model could already outperform the baseline model, in this case, m5UPred. Since the FICC-seq provided the available mRNA sites, we used part of them as inputs in the target domain and trained an additional classifier for direct mRNA m^5^U prediction. Therefore, RNADSN can provide two forms, according to different application scenarios.

### 2.2. RNADSN Outperforms Baseline Models

The performance of a machine-learning model is greatly affected by the input information. Therefore, in our case, the first step in the model evaluation as to determine the optimal sequence length in the input. As the baseline model to be compared, m5UPred used a 41-nanotesla-length RNA sequence with the target uridine centered. Therefore, we tested whether longer sequences could improve the model performance. Specifically, in addition to the support vector machine (SVM) used in m5UPred, we also chose Naive Bayes, Logistic Regression, KNN, Random Forest, and XGBoost as additional baselines. These are the most popular machine-learning classifiers in RNA modification and have been widely adopted for various site predictions [10,13,19]. All of the baselines were run with scikit-learn of version 1.0.2 (https://scikit-learn.org/, accessed on 1 July 2022) and XGBoost Python Package of version 0.9.0 (https://xgboost.readthedocs.io/, accessed on 1 July 2022). Their parameters were delicately tuned to ensure their high performance. The data from the source domain were used as the training data, while the test performance on the target domain was reported for comparison. As shown in Table 1, increasing the sequence length did not improve the model performance in most cases. Therefore, we also used 41-nanotesla-length sequences in our model.

**Table 1:** Accuracy under different baseline models using different sequence lengths.

	41-nt	51-nt	61-nt	71-nt
Naive Bayes	0.7808	0.7766	**0.7873**	0.7846
Logistic Regression	0.6157	0.6210	**0.6254**	0.6216
KNN	**0.4466**	0.4177	0.3871	0.3728
m5UPred (SVM)	0.8438	0.8401	**0.8440**	0.8313
Random Forest	**0.9355**	0.9334	0.9326	0.9310
XGBoost	**0.8816**	0.8796	0.8759	0.8783

Note: Bold text indicates the best performance of each model. All methods were evaluated on the same test and training data (only data length varies) as the source-only case in Table 2.

**Table 2:** Model performance using different training data.

Training Data	Model	Acc	Spe	F1	AUC	AP
Source only	NB	0.7808	0.7930	0.6107	0.8070	0.3471
	LR	0.6157	0.6212	0.4778	0.6378	0.1323
	KNN	0.4466	0.4070	0.3945	0.6878	0.1577
	m5UPred	0.8438	0.8602	0.6756	0.8603	0.4675
	RF	0.9355	0.9814	0.7694	0.8983	0.6526
	XGBoost	0.8816	0.8945	0.7342	0.9131	0.6473
mRNA only	NB	0.8325	0.8434	0.6707	0.8558	0.4266
	LR	0.7141	0.7342	0.5348	0.6692	0.1679
	KNN	0.4964	0.4842	0.4093	0.6256	0.1476
	m5UPred	0.9091	0.9579	0.7038	0.8323	0.4105
	RF	0.9163	0.9987	0.5613	0.8663	0.5771
	XGBoost	0.9258	0.9724	0.7450	0.8767	0.5959
Source + mRNA negative	**RNADSN**	**0.9392**	**0.9639**	**0.6748**	**0.9394**	**0.7670**
Source + mRNA	**RNADSN**	**0.9527**	**0.9862**	**0.7019**	**0.9422**	**0.7855**

Note: All methods were evaluated on the same test data with a positive–negative ratio of 1:10. The threshold for accuracy, specificity, and F1 score was 0.5. Acc: accuracy; Spe: specificity; AP: average precision.

We designed three scenarios to demonstrate that our model is able to capture common features and enhance mRNA m^5^U predictions from tRNA data. In the first case, the baseline models were trained on source-domain data only and evaluated on mRNA data to show the direct transfer performance from tRNA to mRNA. In the second case, the model was trained and evaluated on mRNA data to avoid heterogeneity with the tRNA data. In the last case, we used RNADSN to learn from both tRNA and mRNA data. As mentioned in the framework section, RNADSN can have one classifier, which only processes source data (only mRNA negative data were used as target domains to provide mRNA sequence context), or two classifiers, which process source and target data, respectively. All the performances were evaluated using 36-fold cross-validation, and the average results across the folds were reported.

To simulate a real transcriptome context, in which only a small amount of uridine was modified, a test dataset was constructed using a positive–negative ratio of 1:10. For this imbalanced setting, we paid special attention to the average precision (AP), as it does not affect the times at which the model only predicts true negatives. As shown in Table 2, Random Forest and XGBoost showed promising performances when the model was only trained on the source data without seeing the target domain, indicating a common feature between tRNA m^5^U and mRNA m^5^U. Due to the limited data, the models trained on the mRNA data performed worse than the models trained on the tRNA, illustrating the need to leverage tRNA data. Using RNADSN, when only negative data for mRNA were available, the model improved the average precision by 11.44%. This significant improvement demonstrates the ability of RNADSN to separate the heterogeneity of tRNA from the common features of m^5^U. When mRNA m^5^U-positive data were available and used with mRNA-negative data in the target domain, the average precision was further improved, by 1.85%.

To further assess the robustness of our newly proposed model, we further separated the data according to their source techniques (miCLIP and FICC-seq) and source cell lines (HEK293 and HAP1) and examined the cross-technical and cross-cellular performance. As shown in Table 3, when tested by the independent dataset generated from another technique or cell type, RNADSN achieved AUC of 0.8731, 0.8845, 0.9342, and 0.8765 and AP of 0.5853, 0.5724, 0.7369, and 0.5679, respectively, indicating the effectiveness of our model. In addition, we also tested the performance of m5UPred for comparison, which achieved AUC of 0.7910, 0.7654, 0.7983, and 0.7300 and AP of 0.3142, 0.2569, 0.3127, and 0.2498, respectively, by the independent dataset generated from different techniques and cell types. RNADSN therefore outperforms m5UPred in every metric.

### 2.3. Interpretation of RNADSN Allows Motif Mining

The previous sections underscored the performance of RNADSN. To gain insight into the driving features behind these performances and predictions, we applied the Integrated Gradients (IG) [45] method to obtain a contribution score for each input. TF-MoDISco [46] was used to integrate the model preferences for the k-mers in each tested RNA sequence. TF-MoDISco first extracted high-weight k-mers, and then performed clustering and sequence alignment on similar patterns to obtain global motifs.

**Figure 2:**
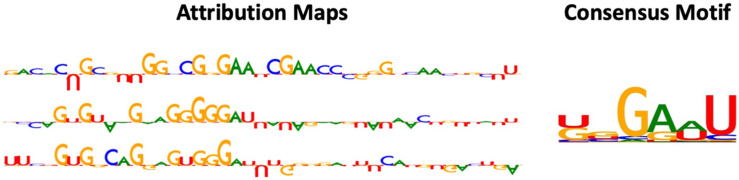
Examples of attribution map and motif detected by RNADSN. In attribution map, upward and downward letters refer to the positive and negative contributions of that nucleotide to the prediction that the central site is a modification. The higher the height, the greater the contribution.

As shown in Figure 2, the motifs captured by RNADSN were similar, but not identical, to the tRNA m^5^U modification motifs reported in the source paper, indicating that our model successfully learned the common features of the two domains rather than tRNA-specific patterns.

## 3. Materials and Methods

### 3.1. Benchmark Dataset

The experimentally detected m^5^U sites were collected from the recently published base-resolution m^5^U sequencing data. The sequencing results were obtained by two technologies (CLIP-Seq and FICC-Seq) on two cells (HEK293 and HAP1). Data were downloaded from Gene Expression Omnibus (GEO), with the accession number GSE109183.

We divided data into three groups, according to their annotation from UCSC human reference (GRCh37), i.e., mRNA, tRNA, and other RNAs. In total, we obtained 457 sites on mRNA, 1076 sites on tRNA, and 2163 sites from other RNAs. In practice, data from tRNAs and other RNAs were merged into the source domain, while mRNA data were treated as the target domain. Negative data were sampled from the same transcript as the experimentally detected sites. For mRNA and other RNAs, the positive–negative ratio was set to 1:10 to mimic the natural distribution of modifications. For tRNA, all experimentally undetected uridines were used as negative data, and the positive–negative ratio was about 1:3. We also separated the collected m^5^U data in terms of their cells (HEK293 and HAP1) and technologies (miCLIP and FICC-Seq) for cross-technical and cross-cellular validation.

### 3.2. Data Processing

Input sequences of varying lengths, from 41 nt to 71 nt, were considered with the target uridine in the center. The input RNA sequences were transformed into numeric vectors through one-hot encoding. That is, A was encoded as (1,0,0,0), C was encoded as (0,1,0,0), G was encoded as (0,0,1,0), and U was encoded as (0,0,0,1).

Since we were interested in the predictive power of the model for mRNA, we split the collected mRNA data evenly into six folds. We fixed one fold of the mRNA data as the test data for all cases (train only on source domain, train only on mRNA data, and transfer learning). The remaining five folds were used as training data in the latter two cases. To avoid training bias caused by imbalanced training data, we further upsampled the positive data in each group to the same amount of negative data. For RNADSN, each time the model receives a source datum, it needs to have a target datum at the same time to learn common features between domains. Therefore, we further upsampled the data in the target domain (mRNA-negative only or upsampled positive with negative) to the same amount as the data in the source domain.

We used cross-validation to avoid the effects of randomness in data partitioning. Specifically, we divided the positive and negative mRNA data into six groups, respectively. Each time, one positive group and one of the six negative groups were taken as test data, and the remaining positive and negative groups were used in training. This yielded 36 combinations, allowing 36-fold validation.

### 3.3. Model-Architecture Design

Our model, RNADSN, borrows and modifies the Domain Separation Network (DSN) framework originally used for image classification, enabling it to generalize for RNA sequence classification. Specifically, we replaced the three encoder modules in DSN with our own networks, which are proven to have the ability to effectively extract features by blending some well-known layers from the 1D convolutional neural network (CNN) and Long Short-Term Memory (LSTM). Thus, given a labeled dataset in the source domain and an unlabeled dataset in a target domain, we can train a classifier on data from the source domain that generalizes to the target domain.

DSN is equipped with models for both private and shared components of the domain representations. The private component of the domain representations is generated for only a single domain, and the shared components are employed for both domains. As shown in Figure 1, DSN has a private target encoder $E_{p}^{t}\left(x;\theta_{p}\right)$ learning to capture components of representation $h_{c}^{t}$ specific to target data $x^{t}$, a private source encoder $E_{p}^{s}\left(x;\theta_{p}\right)$ learning to capture components of representation $h_{c}^{s}$ specific to source data $x^{s}$, and a shared encoder $E_{c}\left(x;\theta_{c}\right)$ learning to capture common features from both source domain and target domain. For each domain, a decoder is trained to reconstruct input samples using private and shared representations so that the input features are separated into the two encoders without loss of information. Only the hidden representations from the shared encoder were used to train the classifier. Two modes of RNADSN were proposed. When mRNA-modification data are not available, RNADSN uses only one source classifier to force the network to learn shared representations capable of distinguishing modifications from experimentally undetected nucleotides. In this case, only mRNA negative data were used in training to provide mRNA-sequence context. When experimentally reported mRNA sites were used in training, we added a target classifier to provide an additional layer of supervision to the model so that the network could also learn mRNA-modification patterns from limited data.

The model was trained by minimizing its overall loss. The loss functions among the outputs of three encoders were employed to supervise the individuality and independence of these three encoders. Specifically, the private- and shared-representation components were pushed apart with soft subspace orthogonality constraints ${ℒ}_{difference}$, whereas the shared representation components were kept similar, with a similarity loss ${ℒ}_{similarity}$. Moreover, to further ensure the validity of the private representations and to add generalizability to the whole model, there was also a reconstruction loss ${ℒ}_{recon}$ for each decoder. Lastly, we also aimed to minimize the negative log-likelihood of the ground truth class ${ℒ}_{target}$ and ${ℒ}_{source}$ for each sample to correctly predict the labels of target and source samples. Combining them together, the final loss function is:

$$ℒ=\;\alpha {ℒ}_{\mathrm{recon}}+\beta {ℒ}_{\mathrm{difference}\;}+\gamma {ℒ}_{\mathrm{similarity}}+{ℒ}_{\mathrm{source}}+\delta {ℒ}_{\mathrm{target}\;}\;$$

where α, β, γ, and δ are weights that control the interaction of the loss terms. These weights were fine-tuned during training and the optimized weights are given in the next section.

### 3.4. Model Training

The first step in model training was to find the optimized hyperparameters for the network. Since RNADSN is a relatively large and complex model architecture, we first trained a simplified network with only one encoder and one classifier by taking all the source domains as training data and the target domain as test data. The layers in the encoder and classifier were the same as those used in RNADSN. After tuning, the final network architecture consisted of one CNN layer with a kernel size of (2, 1) and padding size of 2, one LSTM layer with a hidden size of 8, two BatchNorm layers, and dense layers.

With optimized network hyperparameters, we also tested different weights for loss terms and learning rates when training RNADSN. In our case, we found that learning rate of 0.005, learning rate decay step of 600, step decay weight of 0.9, Alpha weight of 0.02, Beta weight of 0.075, and Gamma weight of 0.25 provided the best performance.

Finally, we performed cross-validation using the training settings determined from the above evaluation. For each fold, we trained for 20 epochs and kept the best model. The reported performance of RNADSN was the average of all fold-evaluation metrics.

### 3.5. Model Interpretation

The above model designs prove and validate the precise performance of RNADSN in predicting mRNA m^5^U sites. Interpreting the logic behind the model’s prediction could shed more light on the mechanisms of the model and further demonstrate its performance. Thus, the Integrated Gradients (IG) method was employed to visually explain what our model values most when making different predictions and obtain the most nucleotides that make the most significant contributions when making positive predictions. By computing the gradient of an output neuron relative to its input, gradient-based attribution methods can reflect the extent to which input features contribute to a particular output through the network. Specifically, the target neuron of interest is each modified classification layer. IG computes the average gradient of output neurons as the input changes along a linear path from the baseline or reference input. It measures the contribution of each input to modification prediction and assigns higher scores to significant nucleotides in the input sequence. To integrate the model preferences of each nucleotide in every single test, the general pipeline carries out high-weight k-mers selection, clustering of similar patterns, and multiple sequence alignment. TF-MoDISco was applied to extract consensus motif from instances with higher-than-average weights.

### 3.6. Evaluation Metrics

Classification performance was evaluated by the area under the ROC (Receiver Operating Characteristic) curve (AUC) and average precision (AP). These two metrics integrate the performance of models under all thresholds (true-positive rate vs. false-positive rate and precision vs. recall, respectively) and can thus more robustly reflect the model’s ability to discriminate between m^5^U and uridine. Widely adopted evaluation metrics, including precision (Pre), specificity (Sp), accuracy (Acc), and F1 score (F1), were also implemented to evaluate predictive performance.

$$Pre=\frac{TP}{TP+FP}$$

$$Sp=\frac{TN}{TN+FP}$$

$$Acc=\frac{TN+TP}{TN+TP+FN+FP}$$

$$F1=\frac{2\times Precision\times Recall}{Precision+Recall}$$

where TP represents true-positive samples, TN represents true-negative samples, FP represents false-positive samples, and FN represents false-negative samples. Generally, the higher the evaluation metrics, the better the model’s performance. However, it is worth noting that when there are far more negative data than positive data (as in our case), the model can achieve a high AUC as long as it can predict sufficient negative data. In such cases, the detection capability of positive data is mainly reflected in the AP.

## 4. Conclusions

In this paper, we introduced a transfer-learning model for predicting mRNA m^5^U data by learning common features from tRNA m^5^U data. The proposed RNADSN architecture treated m^5^U sites from tRNA and other RNAs as the source domain and m^5^U sites from mRNA as the target domain. The network first learns to capture source-specific, domain-specific, and shared representations of the input data and then uses the shared features to perform classification. Specifically, we showed that when only negative mRNA data were given to provide mRNA-sequence-context information, RNADSN already demonstrated a promising performance and outperformed the state-of-the-art method, m5UPred. The performance was further improved when additional supervision was provided using the available mRNA data. We further demonstrated that RNADSN achieved satisfactory results through cross-cellular and cross-technical validation. Moreover, a sequence motif was obtained through the model interpretation and the patterns of the learned common features were revealed. Future work will include applying RNADSN to other biological data for cross-RNA analysis.

## Figures and Tables

**Table 3:** Model performances using different training data.

	Training	Testing	Acc	Spe	F1	AUC	AP
**RNADSN**	FICC-seq	miCLIP	0.9282	0.9732	0.5476	0.8731	0.5853
	miCLIP	FICC-seq	0.8909	0.9174	0.5105	0.8845	0.5724
	HEK293	HAP1	0.9408	0.9632	0.6873	0.9342	0.7369
	HAP1	HEK293	0.9262	0.9722	0.5343	0.8765	0.5679
**m5UPred**	FICC-seq	miCLIP	0.9011	0.9668	0.6282	0.791	0.3142
	miCLIP	FICC-seq	0.7991	0.8231	0.6088	0.7654	0.2569
	HEK293	HAP1	0.8592	0.9019	0.6396	0.7983	0.3127
	HAP1	HEK293	0.9031	0.9763	0.596	0.73	0.2498

Note: All methods were evaluated on test data with a positive–negative ratio of 1:10 to ensure consistency with previous tests. The threshold for accuracy, specificity, and F1 score was 0.5. Acc: accuracy; Spe: specificity; AP: average precision.

## Data Availability

The raw data used in this study are publicly available in the Gene Expression Omnibus (GEO) database under accession number GSE109183.
